# 基于纳升电喷雾电离直接进样质谱的代谢组学分析方法最新研究亮点

**DOI:** 10.3724/SP.J.1123.2021.08026

**Published:** 2021-11-08

**Authors:** Yinlong GUO

**Affiliations:** 中国科学院上海有机化学研究所, 上海 200032

随着科技进步,医学实践正向精准医学迈进。大规模临床样本的代谢组学分析技术对于精准医学目标的实现至关重要,也是精准医学研究不可或缺的技术手段。大规模疾病代谢组学的研究对象为复杂临床生物样本中数以千计的代谢物,其涉及的样本数量多,分析周期长,对现有分析技术提出了极大挑战。色谱-质谱联用法是目前代谢组学分析的主流方法,但色谱分离速度限制了其在大规模复杂临床生物样本分析中的应用。直接进样质谱(direct injection-mass spectrometry, DI-MS)已成为除传统色谱-质谱联用技术之外非靶向代谢组学研究的另一种选择,其主要优势在于快速获得丰富的代谢物质谱信息,显著提高分析通量。但是DI-MS面临着离子抑制效应导致代谢物检测灵敏度降低和缺少色谱维度信息使得定性定量困难等问题。因此,发展与DI-MS相匹配的高灵敏度质谱数据采集技术和先进的数据分析方法是解决问题的关键。

针对上述难题,近日,中国科学院大连化学物理研究所的许国旺课题组^[1]^提出了一种基于纳升电喷雾电离直接进样高分辨质谱(nanoelectrospray ionization direct injection-high resolution mass spectrometry, nanoESI DI-HRMS)的非靶向代谢组学分析方法,为大规模代谢组学研究提供了一种新策略。该方法采用拼接式分段扫描模式采集一级质谱信息,使用数据非依赖(data independent acquisition, DIA)模式采集多个碰撞能下的二级质谱信息。方法不仅显著提高了检测灵敏度,而且获得了扫描范围内所有代谢物的质谱信息,为后续回溯性分析提供了可能。该方法结合代谢物一级质谱精确质量及同位素分布、二级质谱谱图相似度及母离子和子离子强度相关性等标准,使代谢物的定性准确率高于94%。此外,采用一级母离子结合二级特征碎片离子的方式,对各种代谢物进行定量。对没有干扰的代谢物,采用一级母离子进行定量;对于含有同分异构体的代谢物,采用特征碎片离子进行定量。

方法的创新性主要体现在4个方面:(1) DI-MS提高了分析通量,2~3 min即可完成单个样本的分析;(2) DIA模式提高了代谢物的二级覆盖度,支持数据可回溯性分析; (3) 综合多维度质谱信息的定性策略提高了代谢物注释的可靠性;(4) 特征碎片离子定量法可实现含同分异构体代谢物的定量,方法稳定可靠。截至目前,鲜有nanoESI DI-HRMS结合DIA技术用于代谢组学数据定性定量研究的报道,该方法在获取代谢物一级和二级质谱信息方面具有独特优势,将在大规模人群队列样品的高通量代谢组学研究中发挥积极作用。

另一方面,传统的细胞代谢组学研究通常需要数百万个细胞以实现代谢组的高覆盖。减少代谢组学所需的细胞数量有利于拓展研究领域,如对干细胞、循环肿瘤细胞和从组织获得的数目稀少的原代细胞的研究。数十个细胞的代谢组学研究不依赖群体细胞培养,有利于节约人力物力,缩短实验周期,使得细胞生物学研究更为便捷。因此,开发针对数十个细胞的代谢组学分析方法具有重要意义。基于此,许国旺课题组^[2]^在上述工作基础上,建立了一种nanoESI DI-HRMS拼接式质谱数据采集新方法,该方法基于毛细管微探针的细胞取样和96孔板脂质在线提取,在3 min内可从20个哺乳动物细胞中检测到19类脂质和500多种脂质代谢物。

方法优越的分析性能主要体现在4个方面:

(1) 毛细管微探针可以准确、快速、可重复地采集数十个细胞,适用于数目受限的稀有细胞分析;(2) 细胞直接在96孔板中进行脂质提取,减少了常规预处理方法造成的痕量样本损失; (3) nanoESI DI-HRMS显著降低了离子抑制效应,提高了分析通量;(4) 拼接式质谱数据采集显著提高了检测灵敏度,有助于实现数十个细胞中脂质组的广泛覆盖。截至目前,数十个细胞代谢组学分析仍然十分困难,急需好的方法。该工作发展的方法在细胞取样、分析通量、检测灵敏度方面具有独特优势,将在循环肿瘤细胞等珍稀临床样本的代谢组学分析中发挥重要作用。

该实验室上述两项工作通过采用拼接式数据采集模式的nanoESI DI-HRMS,发展先进的数据处理算法,实现了从20个细胞到大规模人群样品的快速、高灵敏度的代谢组学分析。
